# Clinical Diagnostic Implications of Body Fluid MiRNA in Oral Squamous Cell Carcinoma: A Meta-Analysis

**DOI:** 10.1097/MD.0000000000001324

**Published:** 2015-09-18

**Authors:** Xiujuan Tian, Zhiying Chen, Shaomin Shi, Xianwen Wang, Wanli Wang, Ning Li, Jing Wang

**Affiliations:** From the Gynecology and Obstetrics Department of China-Japan Union Hospital of Jilin University, Changchun 130033, China (XT); and Department of Respiratory Medicine, China-Japan Union Hospital of Jilin University, Changchun 130033, China (ZC, SS, XW, WW, NL, JW).

## Abstract

Oral cancer, predominantly oral squamous cell carcinoma (OSCC), is one of the most leading causes of cancers worldwide. Due to a low 5-year survival rate, highly effective methods for the early detection of OSCC are totally needed. MicroRNAs (miRNAs), as promising biomarkers, can bring insights into tumorigenesis of oral cancers. However, studies on the accuracy of miRNAs detection in OSCC have inconsistent conclusions, leading us to conduct this meta-analysis. The aim of this study was to systematically review the articles investigating the diagnostic value of miRNAs in OSCC.

The PubMed, Embase, Chinese National Knowledge Infrastructure (CNKI), Web of Science were searched (updated to June 11th, 2015) to identify all articles evaluating the diagnostic yield of miRNAs for OSCC. The pooled sensitivity, specificity, and other diagnostic parameters were used to assess the performance of miRNAs assays on OSCC detection. Statistical analysis was conducted by employing the R software.

The present meta-analysis comprised 23 studies from 10 articles, including 598 OSCC patients and 320 healthy individuals, available for analysis. The summary receiver operator characteristic (SROC) curve was plotted. Meanwhile, the pooled diagnostic parameters and the area under curve (AUC) were calculated based on all included studies. The pooled diagnostic parameters calculated from all 23 studies were as follows: pooled sensitivity of 0.759 (95% CI: 0.701–0.809), pooled specificity of 0.773 (95% CI: 0.713–0.823) and AUC of 0.832, which indicates a relatively high diagnostic accuracy of miRNAs in differentiating OSCC patients from healthy controls. Meanwhile, In addition, subgroup analyses were conducted to access the heterogeneity between studies, which is based on specimen (serum/plasma/blood/saliva/ tissue) and ethnicity (Asian/Caucasian).

In summary, our meta-analysis suggests that miRNAs might be used in noninvasive screening tests for OSCC, which needs further large-scale studies to be validated.

## INTRODUCTION

Oral cancer, predominantly oral squamous cell carcinoma (OSCC), is one of the most leading causes of cancers worldwide, with an incidence estimated to be >500,000 annually.^1^ Tobacco, alcohol, and betel use were the main risk factors for OSCC and the main high-risk groups were comprised of older adult males who use tobacco and alcohol.^2^ In spite of the great improvements made in the prevention of oral cancer, the 5-year survival rate for OSCC has remained nearly unchanged, which is up to 50%, in the past few years.^3^ It was inferred that the poor restoration effect was mainly due to the fact that the majority OSCC were diagnosed at late stages, whereas the 5-year survival rate would get much improved if diagnosed at early stages.^4^ Therefore, highly effective methods for the early detection of OSCC are totally needed to reduce the mortality of patients.

Conventional oral examination (COE) is the standard method of revealing OSCC, confirming the clinical suspicion by biopsy and histopathological examination.^2^ Conventional visual cancer screenings for some anatomic locations can be highly accurate. For example, visual inspection of skin lesions for melanoma is extremely effective, whose sensitivity and specificity rates reach as high as 98%.^5^ However, there are several disadvantages of COE. First, the histopathology is a rather slow process, which requires several days to fix, embed, and stain the biopsy specimen before the results come out.^2^ Second, COE has difficulty in accurately distinguishing between progressive and unprogressive lesions and could not accurately detect precancerous lesions, which present in clinically normal mucosa.^6^ Finally, the invasive nature of COE is another obstacle for patients. Moreover, the brush biopsy was introduced as another potential method to diagnose oral cancer. Although the brush biopsy could be performed without scalpel biopsy for the interrogation of clinical lesions in the condition that the level of suspicion for carcinoma under clinical features is pretty low, it is still needed to combine with a scalpel biopsy when an abnormal examine result reported, for it does not provide a definitive diagnosis.^5^

Therefore, it is desirable to develop noninvasive and accurate molecular tests for OSCC diagnosis. It is currently reported that molecular markers, such as the tumor suppressor gene (TSG) p53 protein expression, chromosomal polysomy (DNA ploidy) could be adopted in OSCC diagnosis. These biomarkers were used as adjuncts to routine histopathological examination, which contributes to effective management of potentially malignant lesions (PMLs). However, their routine use was still limited due to the high cost, complexity of tests, and the lack of facilities in some laboratories.^2^

Fortunately, novel biomarker, miRNAs, can bring insights into tumorigenesis of ORCC. Evidences indicated that miRNA probably plays an important role in carcinogenesis.^7^ MicroRNAs (MiRNAs) are 19 to 24 nucleotides long noncoding RNAs, base pairing to the 30-untranslated region of target mRNAs, which negatively regulate gene expression.^8^ A single gene can be controlled by several miRNAs, and it is estimated that approximately one-third of all human genes are controlled by miRNAs.^9^ Recent studies indicated that miRNAs involved in regulation of apoptosis, cellular proliferation, and differentiation.^10^ Therefore, miRNAs of expression profiles are commonly used for molecular classification of cancer, analogically to DNA microarrays and mRNA profiling.^11^ It is also anticipated that each miRNA targets hundreds of transcripts, making miRNAs one of the biggest group in gene regulators families.

Statistics have revealed that miRNAs are resistant to RNase activity and may be universally present in cell-free body fluids and excretions, including serum, plasma, tissue, urine, saliva, bronchial lavage, feces, and so on.^12^ It is reported that miRNAs are extremely stable in serum and plasma samples,^13^ which allows translating into consistent miRNA expression levels among individuals, making blood miRNAs one of the most potential biomarkers.^14^ Studies have shown that miRNAs have been released into the circulation and can be tested in small quantities rapidly and accurately.^15^ As stated above, it suggested that miRNAs involved in a wide spectrum of physiologic and pathologic processes and also proved to be expressed abnormal in many diseases, especially in cancers, such as lung cancer, breast cancer, prostate cancer, hepatocellular carcinoma, pancreatic cancer, and so on.

For OSCC diagnosis, 23 miRNAs were identified and 10 of them were confirmed differential expression through Lu's studies.^1^ Among these 10 molecules, 8 miRNAs (miR-10b, -196a, -196b, -582–5p, -15b, -301, -148b, and -128a) were examined upregulated, and the other two (miR-503 and miR-31) were downregulated. Moreover, Maclellan et al found that 5 miRNAs (miR-16, -7b, -338–3p, -223, and -29a) yielded a result of AUC > 0.8.^14^ Above-listed examples all suggested the possible role that miRNAs may play in diagnosing OSCC accurately. However, there have been inconsistencies in the articles regarding the performance of miRNAs for early detection of OSCC. Reis et al reported that miR-31 yielded a sensitivity of 70.4% and a specificity of 89.5% in plasma, whereas the same biomarker in Liu's study only yielded 80.0% sensitivity and 68.0% specificity in tissue.^9^ New evidence has suggested that differences in sample resource, ethnicity, and miRNA profile may explain these conflict results,^16^ whereas a meta-analysis which focuses on the overall diagnostic accuracy of included studies can provide more precise results and offer more reliable assessments on the performance of miRNAs in OSCC diagnosis.

Therefore, we performed this meta-analysis by comparing samples from patients suffering OSCC with samples from healthy individuals to identify miRNAs’ expression in relation to diagnosis.^14^ In this study, we aimed to validate the overall diagnostic value of miRNAs in detecting OSCC. To the best of our knowledge, no previous meta-analysis is available for this issue.

## MATERIALS AND METHODS

### Ethnic Statement

Ethical approval was not necessary for this meta-analysis.

### Search Strategy

This meta-analysis was conducted according to guidelines for diagnostic meta-analysis. The PubMed, Embase, Chinese National Knowledge Infrastructure (CNKI), Web of Science were searched (updated to June 11, 2015) to identify all articles evaluating the diagnostic yield of miRNAs for OSCC. Without the publication date or language restrictions, we use the search items of (“oral carcinoma” or “oral cancer” or “oral squamous cell carcinoma” or “OSCC”), (“microRNAs” or “miRNAs” or “miRs”), and (“diagnostic value” or “sensitivity and specificity” or “ROC curve”). In addition, the reference lists of relevant reviews were independently searched to obtain additional articles.

### Study Selection

To be eligible for this meta-analysis, studies had to comply with the following inclusion criteria: (1) concern the use of miRNAs for OSCC diagnosis; (2) provide a reference standard for the diagnosis of OSCC; (3) give sufficient data on sensitivity, specificity, and sample size; and (4) consist of >30 patients. Studies were excluded based on the following criteria: (1) studies not conducted on humans; (2) studies not mentioning oral cancer in the abstract; (3) studies without control groups; (4) review articles, editorials, commentaries, and (5) studies without complete data.

### Data Extraction

Two independent investigators reviewed the full texts of included articles and recorded the following data: (1) basic characteristics of studies, including the first author, publication year, country, male ratio, source of control, cancer type, miRNA profiling type, sample type, methods of miRNAs detection; (2) diagnostic outcomes, including sensitivity, specificity, true positive (TP), false positive (FP), true negative (TN), and false negative (FN).

### Quality Assessment

The qualities of studies were assessed independently by 2 reviewers using Quality Assessment of Diagnostic Accuracy Studies (QUADAS-2) criteria. The QUADAS-2 tool contains 4 key domains: patient selection, index test, reference standard, and flow and timing, which gives a maximum score of 7.^17^ This tool is especially used to evaluate the quality of diagnostic accuracy of included studies.

### Statistical Analysis

We performed all statistical analyses using R software. The bivariate meta-analysis model was used to summarize the sensitivity, specificity, respectively together with their 95% confidence intervals (95% CIs). The sensitivity and specificity of all included studies were used to plot a summary receiver operator characteristic (SROC) curve and the area under the SROC curve (AUC) was calculated. Similarity between studies was evaluated by chi square and I-square statistics, indicating whether the heterogeneity is significant. A *P* value was < 0.1 or an *I*^2^ was > 50%, which indicated the presence of substantial heterogeneity.

## RESULTS

### Search Results and Characteristics of Studies

**FIGURE 1:**
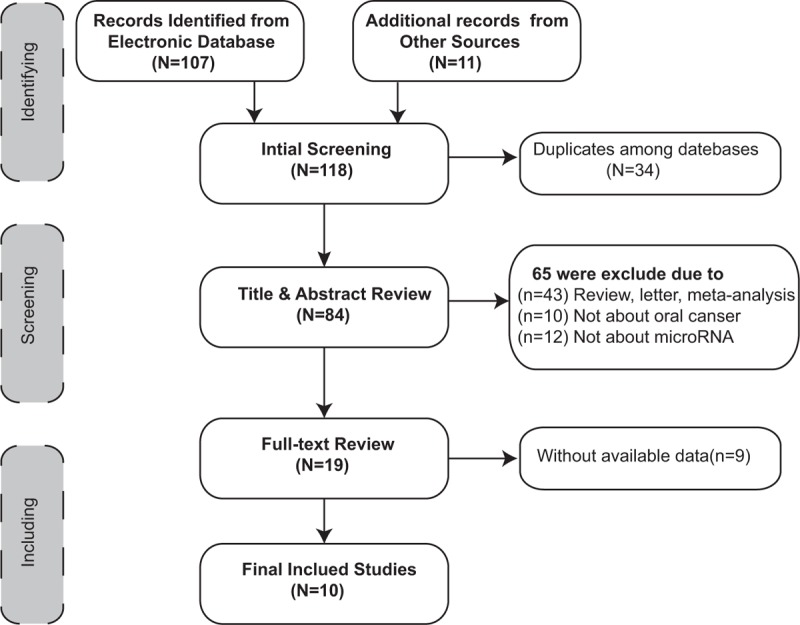
Flow diagram of the study selection process.

The results of our literature research are shown in Figure 1. A total of 118 records were first identified by electronic database (n = 107) and additional records from manual search (n = 11), of which 34 were excluded for being duplications among database. When titles and abstracts were reviewed, 65 were excluded (43 were reviews and letters, 14 were not about oral cancer, and 12 were not about miRNAs), leaving 19 articles for further full-text review, following which 9 manuscripts were excluded due to lack of available data. Finally, 10 articles were included in this meta-analysis.

The present meta-analysis comprises of 23 studies from 10 articles, including 598 patients with OSCC and 320 healthy individuals. Table 1 summarized main characteristics of the included articles in published-year order, ranging from 2010 to 2015. Studies were performed in the following geographical regions: China, Saudi Arabia, Canada, Germany, and Hungary. All included studies focused on single miRNA. The overall studies used quantitative real-time reverse transcription-PCR (qRT-PCR) method to measure the expression of miRNAs. Quality assessment results were shown in Table 1 by using QUADAS-2 evaluation tools.

**TABLE 1:**
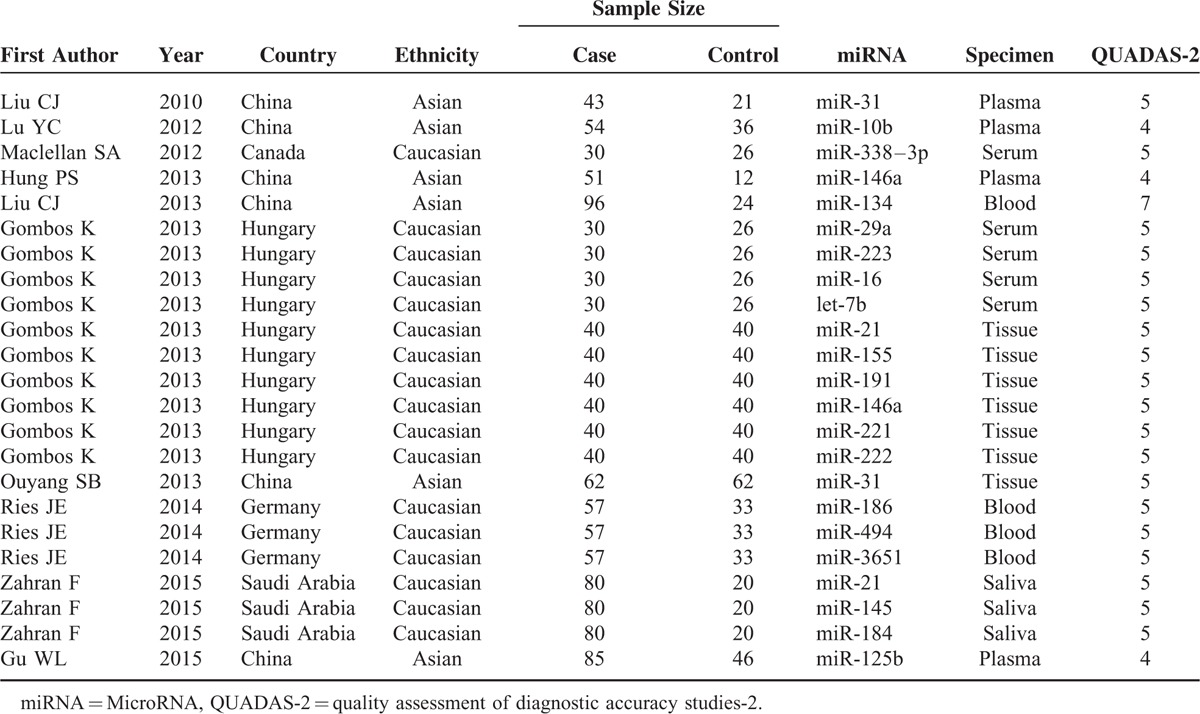
Characteristics of Included Studies in this Meta-Analysis

### Pooled Diagnostic Performance

**FIGURE 2:**
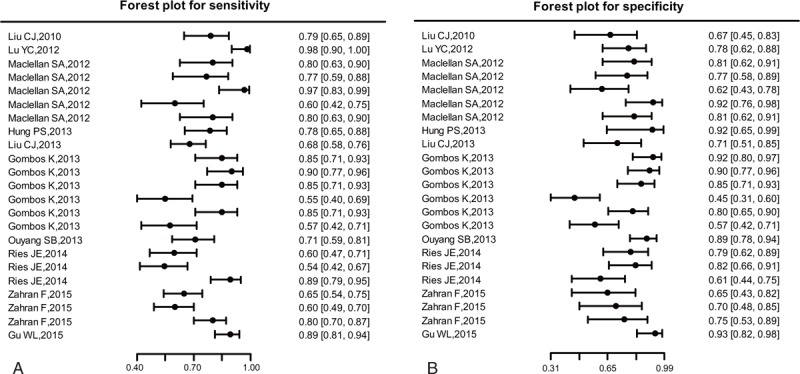
Forest plots of estimates of sensitivity (a) and specificity (b) for miRNAs assays in the diagnosis of OSCC among the included 23 studies. OSCC = oral squamous cell carcinoma.

**FIGURE 3:**
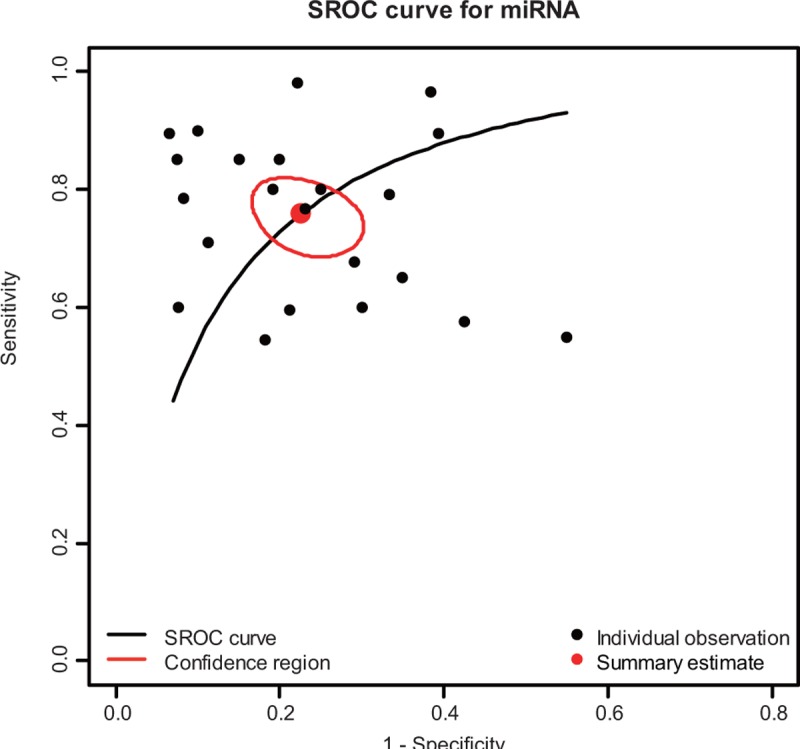
SROC curve for miRNAs assays in the diagnosis of OSCC among the included 23 studies. miRNAs = MicroRNAs, SROC = summary receiver operator characteristic, OSCC = oral squamous cell carcinoma.

Forest plots of data from the 23 studies of single miRNA on the sensitivity and specificity profiling in diagnosing OSCC were shown in Figure 2. *I*^2^ values for sensitivity and specificity were 79.66% (*P* < 0.01) and 70.12% (*P* < 0.01), respectively, indicating that statistical heterogeneity existed between studies. Hence, the random-effects model can be used. The parameters calculated from all 23 studies were as follows: sensitivity, 0.759 (95% CI: 0.701–0.809); specificity, 0.773 (95% CI: 0.713–0.823). In addition, we plotted the SROC and calculated the AUC of 0.83, which indicates a relatively high diagnostic accuracy of miRNAs in differentiating OSCC patients from healthy controls (Figure 3).

### Subgroup Analyses

**FIGURE 4:**
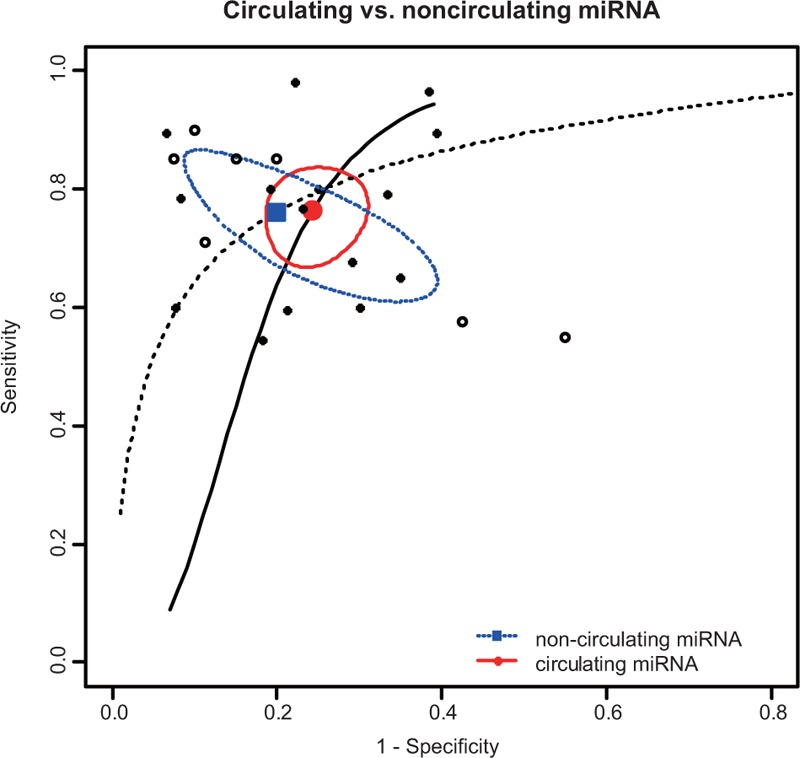
SROC curve for miRNAs assays in the diagnosis of OSCC in specimen subgroups. miRNAs = MicroRNAs, SROC = summary receiver operator characteristic, OSCC = oral squamous cell carcinoma.

**FIGURE 5:**
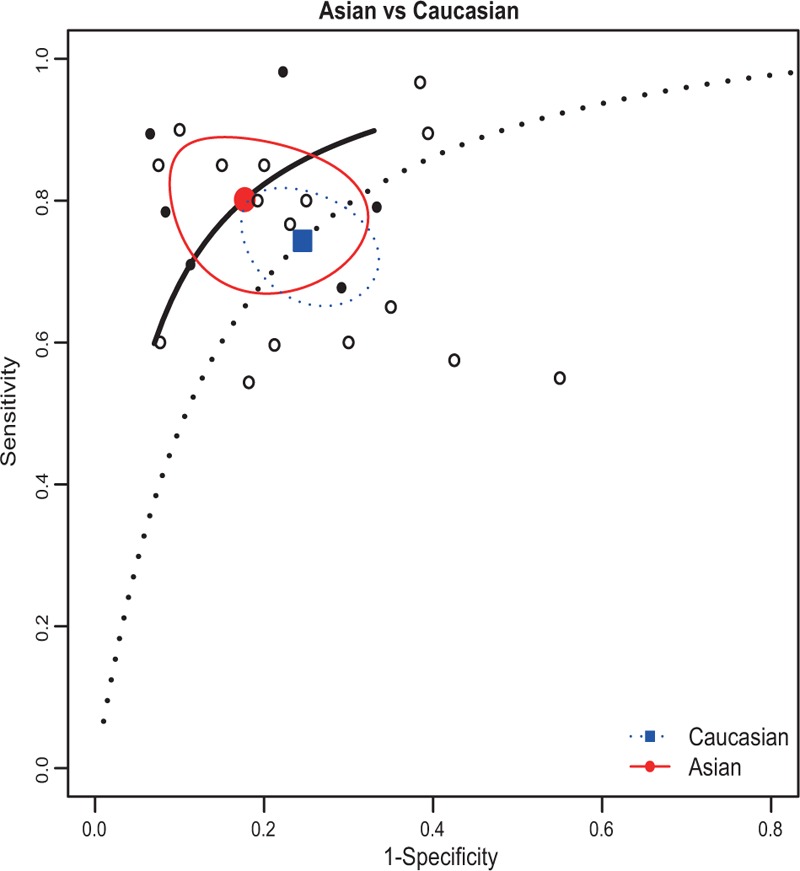
SROC curve for miRNAs assays in the diagnosis of OSCC in ethnic subgroups. miRNAs = MicroRNAs, SROC = summary receiver operator characteristic, OSCC = oral squamous cell carcinoma.

Subgroup analyses were conducted to assess the source of heterogeneity between studies, which is based on the specimen (serum/plasma/blood/saliva/ tissue) and ethnicity (Asian/Caucasian). The SROC curves of sensitivity and specificity for miRNAs in the diagnostic accuracy of OSCC by subgroup analyses were listed in Figures 4 and 5. As for the subgroup of specimen, miRNAs presented a relatively higher sensitivity and specificity in noncirculating group (saliva/ tissue) compared with the circulating group(serum/plasma/blood), with sensitivity of 0.76 (95% CI: 0.643–0.849) versus 0.76(95% CI: 0.688–0.824), specificity of 0.80(95% CI: 0.650–0.896) versus 0.76(95% CI: 0.704–0.803), AUC of 0.84 versus 0.81, and PAUC of 0.799 versus 0.632. In the subgroup of ethnicity, the sensitivity and the specificity of Asian group are 0.80(95% CI: 0.699- 0.875) and 0.82 (95% CI: 0.711–0.898), respectively; the sensitivity and the specificity of Caucasian group are 0.74 (95% CI: 0.671–0.805) and 0.75 (95% CI: 0.684–0.814).

## DISCUSSION

Oral cancer, predominantly OSCC is a leading cause of cancer mortality worldwide, and it remains a low 5-year survival rate for the past years. The accurate and convenient method for detecting OSCC at early stages is necessary. However, existing diagnostic methods for OSCC have their own inherent deficiencies. Take COE for an example: first, histopathology examination of COE is a rather slow process, which requires several days to fix, embed, and stain the biopsy specimen;^18^ second, COE could neither accurately distinguish between progressive and unprogressive lesions nor could it detect precancerous lesions accurately. Furthermore, the brush biopsy must be combined with a scalpel biopsy when the examiner result is abnormal. These facts have compelled researchers to seek reliable, noninvasive, and high-efficiency detection methods. It is reported that miRNAs may play the role as promising biomarkers, with the advantages of high diagnostic sensitivity and specificity and minimal invasiveness. However, the estimation of miRNAs diagnostic performance in OSCC remained inconsistent between studies. In one of such studies, Liu et al reported the high accuracy of miR-10b in plasma for OSCC diagnostic: a sensitivity of 98.0% and a specificity of 78.0%.^7^ Moreover, Maclellan et al identified 20 miRNAs that are significantly deregulated in OSCC patients. In addition, 5 of these 20 miRNAs (miR-16, -7b, -338–3p, -223, and -29a) have an AUC > 0.8.^14^ Nevertheless, Gombos et al found that miR-155 has shown a high sensitivity and an equally high specificity of 90%, whereas miR-146a has shown a low sensitivity of 56% and a specificity of 44%. Due to the inconsistencies of overall studies, we conducted the meta-analysis to determine whether miRNAs could be used as an effective method for early diagnosis of OSCC.

Overall, 10 articles were included in this meta-analysis. The pooled results based on all included studies showed miRNAs yielding an AUC of 0.832 with 75.9% sensitivity and 77.3% specificity in discriminating OSCC patients from healthy individuals. The moderate accuracy of sensitivity and specificity indicates that miRNAs may have sufficient statistical power to confirm or to exclude suspected cases, which enhanced the clinical diagnosis application of miRNAs. Furthermore, the higher value of AUC means better performance of the trade-off between sensitivity and specificity. It is noticeable that the high AUC of 0.832 of this meta-analysis reflects overall relatively high diagnostic accuracy of miRNAs for OSCC diagnostic.

The subgroup analyses were conducted to assess the source of heterogeneity. Specimen (serum/plasma/blood/saliva/ tissue) and ethnicity (Caucasian and Asian) were chosen to perform subgroup analyses. For specimen, the diagnostic performance of noncirculating detection indicates a sensitivity of 0.76, specificity of 0.80, and AUC value of 0.84; and the circulating detection indicates a sensitivity of 0.76, specificity of 0.76, and AUC value of 0.81. The results suggested that miRNAs may serve as a better biomarker in noncirculating group. When focusing on the diagnostic effect of ethnicity, the sensitivity and specificity of Asian group were 0.80 and 0.83, respectively, whereas the sensitivity and specificity of Caucasian group were 0.74 and 0.76. The results show that Asian group has a better diagnostic accuracy than the Caucasian group. The subgroup analyses indicated that miRNAs might be more specific and sensitive in the noncirculating group and Asian.

An interesting finding in this meta-analysis was that we did not include any articles that attached attention on multiple miRNAs with regard to OSCC, which may be a possible factor of the limited clinical effect. As expected, in recent sufficient studies, it indicated that a combination of miRNAs (generally 3–5 miRNAs) was more complete indicators than individual miRNAs in various cancers, such as breast cancer,^19–21^ prostate cancer,^22,23^ colorectal cancer,^24–26^ gastric cancer,^27–29^ lung cancer,^30,31^ and pancreatic cancer.^32^ Therefore, further studies about the comparative diagnosis effect between multiple miRNAs and individual miRNAs on OSCC are needed to be performed.

Although the majority of studies suggested the excellent diagnostic performance of miRNAs, the exact role of miRNAs in the carcinogenesis of tumors remains unclear. MiRNAs may regulate gene expression in the post-transcriptional stage or may generate cellular processes such as developmental transitions, cell proliferation, and apoptosis. Recently, several researchers have demonstrated a functional role for small noncoding RNAs (microRNAs) in the pathophysiology of the cardiac arrhythmia. Santulli et al established a mechanistic association between specific microRNAs and atrial fibrillation.^33^ Meanwhile, Wronska et al provided a systematic overview of the role of microRNAs in the pathophysiology, diagnosis, and treatment of cardiovascular disease.^34^ Therefore, we should perform further studies to clarify the generate mechanisms, which contributes to determine the best miRNAs for future miRNA detection. Furthermore, groups of heterogene tumors are located in the oral cavity, the nasal cavity, and some other locations around,^35^ which have an influence on the biology environment of tumors in regard to OSCC. Thus, the expression patterns of miRNAs in relation to OSCC probably differ from each other because of different tumor locations, which should be treated as an important factor.

Certain limitations in this meta-analysis should be taken into account when interpreting our results. First, although we tried our best to include all relevant studies, it is possible that we may have missed some significant studies in our screening process. Second, all studies were conducted in Caucasian and Asian populations instead of all 3 ethnics, which may produce population selection bias. Lastly, different studies reach contradictory results may be attributed to inconsistent cut-off values. Despite the above limitations, our study is the latest meta-analysis using R software to evaluate the diagnostic values of miRNAs for detecting OSCC.

In summary, the meta-analysis suggests that miRNAs might be used in noninvasive screening tests for OSCC. However, further studies and greater improvements of miRNAs in diagnosis are also needed to validate.
